# Detached from Sympathy, Unconscious of Trauma: The Impact of the Forensic Virtues of Impartiality and Detachment on Rape Examinations in Britain 1924–1978

**DOI:** 10.1093/shm/hkad097

**Published:** 2024-03-12

**Authors:** Pauline Dirven

**Affiliations:** Drift 6, 3512 BS Utrecht, The Netherlands

**Keywords:** sympathy, detachment, impartiality, rape trauma

## Abstract

This article asks why British mainstream forensic literature and practice did not acknowledge the long-term mental consequences of rape for victims and their need for a sympathetic approach before the 1970s. I argue that this was not simply out of ignorance, considering that in the period 1924–1978 there already were some medical practitioners—women doctors, psychiatrists and gynaecologists—who expressed concern for these matters. However, the forensic expert witnesses, who were influential in the field, considered the virtue of sympathy and the practices of care that women doctors promoted to be incompatible with the judicial virtue of impartiality. To avoid any suggestion of partiality, which would damage their authority in the adversarial courtroom, these men instead employed the epistemic virtue of emotional detachment. This led them to adopt a sceptical attitude towards rape victims and drew their attention away from the psychological care women and children might require.

## Introduction

In contemporary handbooks of forensic medicine, authors emphasise the long-term psychological impact rape can have on victim–survivors.^1^ The authors stress that forensic examiners should pay attention to the emotions of the complainant and comport themselves in a ‘sympathetic but professional’ manner.^2^ In the 2020 edition of *Clinical Forensic Medicine,* it says

Throughout all the stages of the clinical forensic assessment, the forensic practitioner must avoid partisanship while remaining sensitive to the immense psychological and physical trauma that a complainant may have incurred. The continuing care of the complainant is essentially an ongoing process throughout and beyond the primary clinical forensic assessment.^3^

Care for victim–survivors and sensitivity to the psychological trauma they might have sustained are primary tasks of the examiner.^4^ Contemporary forensic clinicians should assume a sympathetic attitude while avoiding partisanship.

This is a significant change in perspective. Until 1978, when Burges argued that police surgeons should adopt a sympathetic attitude towards rape victims in the handbook *The New Police Surgeon*, authors of British forensic handbooks cautioned their readers that the expression of sympathy towards rape victims would connote partiality. They considered the British legal virtue of impartiality, to which they had to adhere as expert witnesses, to be incompatible with sensitivity. As I will explain below, this idea caused considerable harm to rape victims. Forensic experts’ primary focus was on the collection of evidence and physical injuries to corroborate the allegation, and not on providing psychological care. They denounced a sympathetic attitude in favour of a detached, sceptical and sometimes even hostile approach and in principle questioned the story of rape complainants because they subscribed to the rape myth ‘women lie about being raped’.

Until the publication of *The New Police Surgeon* in 1978, there was no attention to psychological trauma of rape victims in mainstream forensic literature. This is noteworthy, considering that psychiatrists and psychoanalysts had already started to discuss the impact of heightened emotional states, especially fear, on the nervous system, in the 1870s.^5^ This attention to psychological trauma as a result of an emotional experience increased in Britain in the aftermath of the World Wars. World War I attracted attention to shellshock and the emotional consequences of the war on soldiers and World War II sparked interest in the mental health of citizens, especially children under air raids.^6^ However, as historians of sexual violence argue, these insights into the impact that fear could have on mental health were not applied to rape in the early and mid-twentieth century.^7^ Historian Joanna Bourke argues that ‘medical and psychiatric personnel generally ignored the psychological responses of rape victims until the 1960s—and, even then, it was rare until the 1970s’.^8^

In a similar vein, I found that forensic handbooks did not address the long-term psychological impact of rape nor wrote about the need for practitioners to adopt a sympathetic approach. In contrast to the historiography on rape examinations though, this article proposes to look beyond psychiatric and forensic literature on rape and study medical practices related to rape in other medico-legal cases, such as abortion, and outside the forensic realm. This approach illustrates that knowledge on long-term mental consequences of rape was articulated in the period 1924–1978.^9^ To illustrate this I analyse two case studies: advice of the Medical Women Federation (MWF) to the Home Office on medical examinations in sexual crime cases that started in 1924 and the 1938 trial of the gynaecologist, Dr Bourne. Both are rich sources on the topic of psychological trauma in rape victim–survivors but have received limited attention in the historiography.^10^ The trial of Dr Bourne, who was tried for performing an illegal abortion on a rape victim, demonstrates that the idea that rape could have long-term psychological consequences already surfaced in the courtroom and medical journals in the late 1930s. And letters written to and by the MWF, in the period 1924–26 and 1942–43, illustrate that women doctors were concerned with rape victims’ injuries ‘of the mind’ decades before 1978.

This raises the question why mainstream forensic advice literature on sexual violence in the early and mid-twentieth century did not address the issue of mental harm or advise a sympathetic attitude. I argue, first, that part of the explanation lies in gendered ideas that influenced medico-legal appointment procedures and denied doctors from the MWF the opportunity to impact examination practices. And second that this discrepancy can be explained by expert witnesses’ concern about being critiqued for emotional involvement in a case. The cases of the MWF and Dr Bourne illustrate that medical practitioners associated a physician’s attention to the mental well-being of sexual assault victims with feelings of pity for, or, at least, a display of sympathy towards the victim. And forensic experts feared that such a display of sympathy would be incompatible with their attempts to embody the epistemic virtue of impartiality.

To embody judicial impartiality, forensic experts instead advised one another to apply the modern medical virtue of emotional detachment. This implied taking out of the equation the emotions of both the complainant and the expert. In British forensic culture, such a detached approach was particularly strong as it was strengthened by the British emotional landscape of the ‘stiff upper lip’ and ideals of controlled masculinity, in the male-dominated field of forensic medicine. Before I outline this argument, I first explain how my attention to the modern medical virtue of emotional detachment adds to the existing literature on the unsympathetic attitude of medical staff in rape examinations.

## Hostility in Rape Examinations

Historians studying medical examinations of rape cases, offer several explanations as to why forensic doctors were unsympathetic towards rape victim–survivors. Most point to shortcomings in the institutional organisation of forensic medical services in the British criminal justice system and the impact of rape myths.

First, historians of forensic culture have demonstrated that during most of the twentieth century, Britain lacked a formal system for the employment and education of forensic experts.^11^ Anyone with a medical degree could examine a rape victim–survivor without needing a special qualification in forensic matters in general, or sexual violence specifically. In practice, this meant that the police chose to consult their police surgeons or ask for the help of local general practitioners. Since many of the doctors lacked proper training, the examinations could be painful, vital evidence could be lost and victims did not receive proper psychological care because the practitioners were unfamiliar with the traumatic effects of rape.^12^ Moreover, as Joanna Bourke explains, police surgeons became ‘enmeshed in police culture [and] … aligned themselves more with the law than with medicine’.^13^ Police surgeons only saw the victims once and therefore did not consider them as patients but prioritised the collection of evidence overtreatment, especially over the treatment of psychological wounds.^14^

Second, historians of sexual violence and forensic medicine argue that contemporary gender prejudices and rape myths, especially the myth that ‘women lie about being raped’, influenced forensic examiners. They emphasise that in the courtroom and the office of the medical examiner, gendered behaviour was as much on trial as the actual crime that was being tried.^15^ The complaints of women who did not conform to ideals about femininity, such as women who had willingly engaged in sex before marriage, were likely to be discarded. Since many doctors accepted the myth that most women lie about rape, they adopted a sceptical attitude towards complaints and saw it as their job to try and unmask these women. Such as ‘women who have become frightened by what they have possibly willingly allowed to take place, by women who are pregnant and hope that the police may find the father, by women hoping to gain notoriety from their accusations, and by women who have found themselves in a difficult position as a result of their actions’.^16^

Joanna Bourke offers a third explanation of why forensic doctors lacked sympathy for rape victims. She argues that police surgeons were emotionally affected by their encounters with rape victims. According to her, ‘by witnessing and examining the appropriated and wounded body of a victim of sexual violence, the police physician also “hurts”’.^17^ To cope with this kind of wounding, she suggests that police surgeons adopt coping mechanisms, especially a cool professional attitude.

In this article, I want to explore in more detail the context in which forensic experts adopted such a detached or ‘cool’ attitude. I illustrate that acting in a detached manner was not merely a coping strategy police surgeons employed but a performance of forensic expertise that was deeply embedded in the intersection of the medical, legal, social and gendered culture in which they operated. To be more specific, forensic physicians adhered to the modern medical virtue of detachment—strengthened by gender norms and the British stiff-upper-lip—as it complemented the judicial virtue of impartiality.

Social histories of medicine show that the development of a more scientific, team-based and specialised form of medicine in the early twentieth century, changed physicians’ ideas on whether and how they should display sympathy for their patients.^18^ Many medical practitioners opted to adopt ‘detached concern’, which meant that they sought to abolish emotional reactions from their relations with patients. Others criticised this shift away from an empathic approach and advocated a professional display of empathy, which allowed doctors to ‘feel into the pains’ of their patients while still distancing themselves from the patient’s problems.

To understand why the detached, and even unsympathetic, approach had the upper hand in rape examinations this article considers how medical ideals coincided with judicial virtues. Forensic expert witnesses operated at the crossroads of medical and legal cultures. During the early and mid-twentieth century, forensic services in Britain were slow to formalise and institutionalise. Forensic physicians were actively attempting to carve out a position for themselves as trustworthy and valuable knowledge-makers in the criminal investigation process.^19^ To accomplish this in an adversarial justice system, they strove to perform the epistemic ideal of impartiality, not only in the courtroom but also in the examination space. This attempt to embody the judicial virtue of impartiality let forensic doctors devalue the medical ideal of sympathy as they feared attention to emotion connoted bias. Instead, they opted for the virtue of detachment. This approach caused them to neglect the psychological needs of victims or consider their mental anguish—if they acknowledge it at all—solely as a piece of evidence and not as a matter that required empathy or care. This is remarkable considering that knowledge of the long-term psychological consequences of sexual crime and the necessity of a sympathetic approach was already advocated in interwar Britain, as the next two cases will illustrate.

## ‘To Implant in the Mind Seeds of Terror’

The 1938 case of Dr Aleck Bourne has received little attention in the forensic historiography, and then mostly for its impact on abortion law. However, if we shift the perspective from the outcome of the case to the in-depth discussion among the expert witnesses during the trial, it shows that some gynaecologists and psychiatrists already discussed the psychological consequences of rape in the interwar period.

On 18 and 19 July 1938, Bourne stood trial for performing an unlawful abortion under the Offences Against the Person Act of 1861. He had aborted the foetus of a 14-year-old girl who had become pregnant as the result of rape by three soldiers on 27 April 1938. In his reflections on the case, Bourne claims that he wanted this case to come to trial so that the court would be forced to consider the question of whether the danger to mental health was sufficient reason to produce a legal abortion.^20^At the time, it was accepted that an abortion would be legal when performed by a medical practitioner to save the mother’s life but Bourne’s defence was that, even though the mother’s life was not in direct danger, the abortion had been lawful because her health was in danger. In the *British Medical Journal,* Bourne wrote that he wanted to imprint on the law that mental health was just as important, or even more important, than physical health.^21^ He clarified that, in addition to her youth, the deciding factor for terminating the pregnancy was ‘the fact that she, a virgin, had been raped’.^22^

The rape had not yet been proven in court when Bourne carried out the operation on 14 June; it was not until 28 and 29 June that the soldiers were convicted of rape in the Central Criminal Court. Moreover, in this case, as in so many other rape cases, the character of the girl was questioned throughout the rape trial. During the last days of the trial, the defence attorney emphasised that she had worn lipstick and powder and that she could have known that one of the soldiers, at least, wanted to kiss her.^23^ The judge, in his summing up, also remarked to the jury that they might think that the girl looked older than her age and that ‘a good many girls are quite prepared for a flirtation’.^24^ Despite the use of these rape myths to question the girl’s statement that the act had not been consensual, Bourne did not seem to be in doubt that she had been raped and was suffering from the consequences when he saw her on 14 June. As I will argue below, this was in stark contrast with the sceptical approach that was advocated in handbooks on forensic medicine.

Bourne based his assessment of the girl’s mental health on her story. When he saw her a month after the attack, he concluded she was still suffering from the consequences. Bourne had carefully observed the girl’s emotions in the hospital. According to the ward sister the girl had been unusually cheerful but Bourne believed that this was a ‘façade of courage erected as a defence against her feelings’. He carefully watched her demeanour when he took swabs to test for infection and V.D. and ‘instead of her bearing the trifling discomfort with fortitude, she had broken down and cried. That had confirmed his decision and later in the day he had operated’.^25^ According to Bourne, this rape already had a long-term—month and a half—impact on the mental health of the girl.

Moreover, Bourne believed that the rape could have even more long-lasting psychological consequences for the girl if she would carry the pregnancy to term. Bourne argued that this ‘would have been a source of nervous, psychoneurotic, and other troubles’ because ‘the circumstances of her conception were … such as to implant in the mind seeds of terror’.^26^ He reasoned that mental symptoms would arise because the girl had conceived the child in—what he assumed had been—a state of terror. His decision for a medical intervention was thus based on the knowledge that her nerves would be affected by the experience of rape and the resulting pregnancy.

Bourne portrayed himself as an advocate for mental health in child rape victims during his trial and his media performance afterwards when he was acquitted. His defence had hinged on the idea that emotional well-being, mental health and rape were integrally connected. Several of Bourne’s colleagues had supported his decision at the time of the operation and during the trial expert witnesses agreed that terminating the pregnancy was the right decision because the girl’s ‘mental condition would be such that she might be in grievous danger, and that her nervous system stood a good chance, or might stand a good chance, of being shattered’.^27^

Historians illustrate that in Britain in the early twentieth century, psychologists, psychiatrists and psychiatric social workers already paid attention to child psychology and psychoanalytical ideas concerning trauma and childhood behaviour.^28^ Bourne’s trial shows that they also applied psychoanalytical ideas to cases of sexual assault. One expert witness, psychiatrist John Rawlings Rees, compared the mental results of the rape in combination with the pregnancy with the ‘shell-shock’ soldiers developed during the Great War. He was close to certain that the girl would suffer a mental breakdown. He had two patients in his care who had become pregnant in similar circumstances and had carried their babies to term, and they ‘suffered from varying neurotic difficulties which had crippled them; neither had made a successful marriage, both were terrified at all matters connected with sex, and were anxious and unstable in every way’.^29^ He even had one patient who had developed schizophrenia.^30^ When asked whether it was possible to distinguish if these symptoms were the result of the rape or the pregnancy Rees explained that the mental symptoms were grave when this unique situation presented itself. His most important conclusion was that in general, for victims of sexual offences, ‘the dramatic episode was not so important as the continued strain and the general emotional atmosphere’.^31^ Because Rees testified in favour of performing the abortion, he emphasised the specific mental impact of pregnancy resulting from rape rather than reflecting substantially on the long-term psychological effects of the rape event alone. Still, when asked about the possibility to differentiate between the impact of the rape and the resulting pregnancy, he admitted that the data he had collected about cases of rape where conception did not take place, showed that ‘symptoms might certainly appear in such cases’.^32^ He saw the long-term consequences of both a pregnancy resulting from rape and rape in which conception did not take place in the 1930s.

This example shows that decades before the 1970s, there were medical practitioners who believed that sexual offences caused long-lasting mental suffering in child victims of rape and that they referred to it in a psychological discourse, of shell shock, schizophrenia and neurosis. Bourne and the expert witnesses were not the only ones who considered the long-term psychological effects of rape on children.

## The MWF on Sympathy and Psychological Trauma

Psychological trauma and long-lasting emotional consequences of rape were topics of discussion for some, predominantly female, general practitioners, police surgeons and psychotherapists. This is clear from two files containing correspondence from the Medical Women’s Federation (MWF) held at Wellcome Collection.

The first file is a set of letters written between 1924 and 1926. In 1924 the Home Office (HO) set up a committee to ‘enquire into sexual offences against young children’ but neglected to include women doctors on the committee. The MWF wrote the secretary of the HO expressing their regret that no women doctors were appointed and urging them to reconsider this decision. That did not happen but the committee did contact the MWF and asked its members for suggestions to strengthen or improve the existing law on or procedure for assaults on young persons. The MWF collected evidence and made various recommendations, especially regarding the employment of sympathetic female police surgeons. Although the committee indeed put forward the recommendation that ‘where a medical examination of a girl is necessary it should, whenever possible, be carried out by a woman doctor’, not much changed.^33^

Therefore the MWF pursued this issue further in 1938 and 1942. In 1938, they drew up a resolution in which they ‘urge[d] that every Chief Constable or Watch Committee should appoint one or more medical women’ for the purpose of examining ‘women and children involved in cases of indecent or criminal assault’.^34^ They believed that their sympathetic approach could lessen the emotional strain on these victims. In 1942 they sought support for this resolution from the National Federation of Business and Professional Women’s Clubs of Great Britain and Ireland before they brought it to the attention of the HO again. The second file contains these letters.

The correspondence between members of the MWF and National Federation, which has not yet been analysed in depth by historians of sexual violence, illustrates that already in the 1920s and 1940s, this community discussed the impact rape and forensic examinations had on the mental health of victims of sexual crime. The historiography of rape trauma suggests that psychiatric texts did not consider the development of emotional disorders after sexual assault before the 1970s, and on the rare occasions that they did, focused solely on the emergence of frigidity.^35^Historians studying medical practices, instead of textbooks, have illustrated that even though long-term mental consequences were often absent from medico-legal practices, physicians and lay persons did discuss the shorter-term emotional impact of rape, referring to signs they observed hours or days after the attack.^36^ The correspondence of the MWF shows that in the 1920s and 1940s these women doctors already recorded a variety of mental consequences of sexual violence years after the attack in their practice.

For example, Dr Evie Evans, who ran a V.D. Clinic in Cardiff, wrote in 1925 that in her practice she had encountered a few cases of women and children who had been assaulted and suffered from it well after the event. She referred to

a young woman patient of mine whose outlook in regard to the question of accepting a certain man in marriage was very much poisoned by the recollection of a friend of her father who had taken her on his knee as a little girl and had sexual contact with her. Her mind had certainly been injured though her body was not.^37^

This ‘injury of her mind’ impacted her life years after the abuse had occurred. A similar case was reported in 1942 by Dr Elspeth Macleod, of the Institute of Child Psychology, who referred to the case of Mrs B who had ‘a terrifying sex experience at the age of four years’ and suffered from the ‘frightening fantasies’ surrounding the event for 30 years.^38^ She explained that this gave the victim emotional distress and nervousness to the extent that she had a ‘fear for going out alone’, longed ‘to lie in bed and do nothing’, and was close to a ‘complete mental breakdown’.^39^ A similar view on the long-term emotional impact of rape was uttered by the first female police surgeon to be appointed in Britain in 1927, Nesta Wells. She published her ideas in the *British Medical Journal* in 1961, where she stated

there are many rather sensitive, often intelligent girls who never seem able to see things again in quite the same way. Their attitude to boys and men will never be quite natural, unreasonable fears of many kinds may develop, and their whole emotional life may be thrown out of balance. The sooner these girls get psychiatric help the better.^40^

At different times in the period 1924–1961, various women physicians highlighted that sexual violence had long-term mental consequences, especially when children were concerned.

It is important to note that, even though the 1942 circular of the MWF referred to cases involving both women and children, most female doctors limited their attention to child victims. In the medical profession in general, female professionals had, since the interwar years, shifted their attention from adult women to children.^41^ While some doctors stressed that both women and children should have the right to be seen by a women police surgeon, others did not consider the case of adult victims and the secretary of the York Diocesan Association for Moral Welfare even claimed that in her experience ‘girls over 16 prefer to be examined by a man but it is real anxiety to girls under 16 to be examined by a man’.^42^

The MWF’s focus on children aligned with the research interests of British psychoanalysts who, during the 1920s and 1930s had a major ‘interest in child psychoanalysis’.^43^ During the Second World War these professionals focused on the mental health of children during aerial bombardment.^44^ Lastly, the sole attention to children’s mental well-being might also be influenced by Freudian understanding of trauma. Indeed, Freudian understandings of psychological disfunction in adulthood ‘were heavily biased towards aetiologies of trauma located either within infancy and early childhood or within the unconscious’.^45^ However, the correspondence does not explicitly refer to Freud’s legacy.

According to Dr Macleod, the use of a sympathetic woman doctor could help reduce the mental distress of the rape victim. In their attempts to establish a position for women in forensic investigations, several doctors from the MWF suggested that the virtue of sympathy was related to the gender of the police surgeon. Historian Louise Jackson has illustrated that women police officers and police doctors during the interwar years argued that work with women and children was ‘more appropriate for women than for men by virtue of their sex’.^46^ Her research shows that in the police force, various professional women attempted to carve out a position for themselves by emphasising that women possessed ‘natural sympathy and understanding’ and ‘that they were more likely to get an accurate account of events because of their ability to gain the trust of women and children’.^47^ In general medical practice, women doctors were known for their empathetic relationships with their patients.^48^ In 1942, women doctors writing to the MWF added to these arguments that their gender was less likely to cause mental distress and trauma to the victim. In 1942, Dr Mabel Lindsey argued ‘if a man doctor is called to see the child, no matter how nice or sympathetic he may be in making his examination it may well seem to the child a mere repetition of a terrifying experience received from some other man, as the examination involves looking and feeling the private parts’.^49^ These medical women pointed to the presence of psychological strain in rape cases to justify the role they wanted to play in forensic examinations.

Not all doctors writing to the MWF were convinced that the services of a sympathetic woman doctor would be enough to protect the mental health of victims.^50^ They emphasised that to prevent ‘neurosis’ all victims of sexual crimes should receive skilled psychological care. MacLeod explained that ‘the emotional results of any abnormal sex experience may be so grave that the child may develop symptoms of maladjustment of such a character that his or her life is ruined, by ill-health, neurotic attitude, or anti-social behaviour’. The necessity for psycho-therapy was also mentioned in a 1942 letter by Miss Beattie from the Edinburgh Women’s Business Club who said that by only requesting that women police surgeons be appointed ‘our president [Dr Chaplyn] does not think you are going far enough by and would have a Psycho-Therapist consulted too, but we decided last night to go forward one step at a time’.^51^ The Federation agreed to the latter and in their letter to the Secretary of State for Home Affairs, they did not mention the need for specialised psychological care but only mentioned the ‘psychological’ importance of making sympathetic women police surgeons available to victims of sexual crimes.^52^ The emphasis that these different women doctors placed on psychological care illustrates their awareness of the long-term mental impact sexual violence had on children.

In sum, the letters of the Medical Women’s Federation and the case of Dr Bourne show that, in the first half of the twentieth century, there were medical practitioners who considered the long-lasting mental impact of rape and pleaded for a sympathetic approach towards victims to reduce their mental strain. The next section will illustrate that these ideas were not included in mainstream forensic teachings and practices.

## Emotions in Mainstream Forensic Literature and Practice

In the period 1924–1978, forensic handbooks and lectures by forensic experts did not mention the MWF’s advice to the HO nor the views expressed in the Bourne case. But they did refer to emotions related to rape cases, especially the emotion of fear. In this section, I argue that forensic physicians’ motivation to look for signs of fear did not come from a medical concern for the complaint’s well-being but from their attempt to find evidence that fitted the legal interpretation of rape. For them, fear was a piece of evidence.

Before the Sexual Offences (Amendment) Act of 1976, rape was defined in the Offences Against the Person Act of 1861 as unlawful ‘carnal knowledge’ of a woman by force and against her will of a male of 14 years or older. Force was primarily interpreted as physical violence and the woman was expected to ‘struggle to her uttermost … in an attempt to prevent the commission of the act’ to show that it was ‘against her will’.^53^ Because of this, medical evidence hinged first and foremost on physical injuries that suggested that the woman had resisted. It was not always necessary that the complainant had sustained injuries to prove rape because ‘the force used [could also have been] moral and not physical—for example, threats, fear, horror, syncope’ and if a woman would ‘yield through fear or duress it [was] still [considered] rape’.^54^ Most manuals on forensic medicine emphasised that an experience of fright could explain the absence of physical injuries.

For example, professor of forensic medicine, John Glaister Jr remarked in his 1942 handbook, that ‘there are, unquestionably, girls who become panic-stricken when an attack of this kind is made upon them, and are rendered incapable of offering serious resistance’.^55^ In forensic literature the crime of rape was associated with an emotional response; terror, which the authors believed could have a physical manifestation: paralysis.^56^In 1926, professor of forensic medicine, Sydney Smith explained

Inability to resist from terror or from an overpowering feeling of helplessness, as well as horror at her situation, may lead a woman to succumb to the force of a ravisher without offering that degree of resistance which is generally expected of a woman so situated. … One has to only to consider how in a sudden emergency any one of us may be temporarily paralyzed, to understand the effect on a woman suddenly accosted by a man whose intentions are obvious.^57^

Here, Smith tried to convince his—primarily male—readers of the possibility that women could be paralysed by fear by letting them reason from their own bodily experiences in frightening situations.

Because the law only required adult women and girls who looked as if they were 16 or older to prove intercourse had been ‘against their will’, most handbooks discussed fear only in cases of adult and teenage victims. Moreover, as most forensic doctors assumed that rape of children would normally produce visible injuries even when no additional manual force was used, they did not typically look for other evidence of rape than physical signs in these cases.^58^ The absence of references to the emotions and psychological impact of rape in cases where children were concerned, underlines that the authors of the handbooks considered fear as a piece of evidence and not a matter that required psychological care.

The argument that terror could render a woman, or a girl paralysed put forensic doctors, whose job it was to look for physical signs of rape, in a difficult position. What were they to comment on if no injuries were visible? Authors of handbooks explained that in these instances, the doctor should look for proof that the complainant had felt fear.^59^

Despite the importance that the authors placed on signs of fear, neither Glaister nor Smith nor any of their colleagues explained how physicians should interpret the mental state of a complainant. In the early and mid-twentieth century, it was uncommon for authors of the handbooks to mention the long-lasting psychological effects of rape or to include references to psychological theories. These authors had a background in medicine rather than psychiatry or psychology, and even though the British medical establishment had started to value the knowledge of psychology and psychoanalysis in the 1920s and 1930s, the majority of practitioners did not accept the Freudian approach that focused on sex and emotions.^60^

The authors of the handbooks did not express any concern about the mental health of the victims in the aftermath of the crime. They approached the emotional impact of rape as a short-term manifestation of fear during the attack. That authors were interested in a fleeting emotional state, is underscored by their claim that it was difficult to assess the emotional experience of the victim because days sometimes passed between the rape and the medical examination.^61^

To assess in retrospect whether a victim had experienced fear, some physicians maintained that they could find evidence of this on the body. For example, C. F. Patterson, an expert witness in a violent carnal knowledge trial in 1931, underpinned his observation of anxiety and stress in the complainant by pointing to her rapid pulse.^62^ And Keith Mant argued in 1960 that ‘examinations in cases of [rape through fear and paralysis] frequently reveal much more severe injuries to the genital region than one would expect from sexual intercourse by consent’.^63^ These were exceptions though, as the authors of forensic texts rarely linked emotions to bodily signs.

More often forensic experts stepped outside the medical realm to assess whether a complainant had experienced fear. Commonly they applied lay methods for determining the emotional effects of rape on different kinds of women; especially assessing the moral character of the victim to decide whether it was probable that she had felt fear. Forensic experts warned their readers that anxiety and ‘physical paralysis through fright’ were often feigned by women and girls ‘to save their character’.^64^ Keith Simpson argued in his 1947 handbook that ‘no difficulty is experienced in singling out the chaste from the wanton, or the shy and bewildered from the brazen and affectedly hurt’.^65^ Throughout the period 1920–1960, the handbooks instructed their readers that when the victim was ‘young and inexperienced, physically frail or nervous, she is the more likely to have been taken by surprise and overcome than when experienced in such matters, strong and able to give as good as she gets’.^66^ Only a select group of victims, predominantly virgins and middle- or upper-class women, were believed to have experienced fear.^67^ The experts did not rely on research when they put forward these ideas but instead reasoned from dominant lay norms on gender, sexuality and class to argue that ‘delicately built’ virgins were capable of genuine physical paralysis because of their ignorance of sexual matters. They expected other girls, who were supposed to be aware of the perpetrator’s intention such as girls who had had sexual intercourse before or ‘country girls’ who were ‘familiar with animal life’ to resist.^68^

These statements reveal that the authors assumed that women would become paralysed by fear because of their ignorance of sex and not because the experience of rape was a source of horror or mental distress. By putting forward this idea, forensic experts reinforced the rape myth that only virgins were genuine rape victims and disregarded the idea that the experience of rape was a source of psychological strain in itself.

The link that the authors of forensic handbooks drew between morality and emotions led them to suggest that emotions played a role in rape cases only on rare occasions. All handbooks highlighted that false accusations of rape were frequent and that it was the duty of the medical practitioner to ‘unmask’ lying women. This encouraged forensic doctors to adopt a sceptical attitude when they approached a complainant instead of a sympathetic approach.

It is difficult to determine to what extent this advice from the handbooks on the role of emotion in rape cases played an important part in forensic practice. Because of privacy regulations, there are only a few trial transcripts and depositions of rape cases open for review at the National Archives. These include cases tried at the Court of Criminal Appeal and the Central Criminal Courts for criminal cases in London, Middlesex and parts of Essex, Kent and Surrey. Four of the 33 transcripts and depositions of trials that are open for review and contain medical testimonies suggest that, at least occasionally, the advice to note down short-term emotional responses of rape victims was put into practice. But in none of these files did medical witnesses refer to long-term psychological disorders. Trial transcripts from 1928, 1931, 1959 and 1960 illustrate that expert witnesses mentioned, for example, that the complainant ‘suffered from shock’, appeared ‘anxious and distressed’, ‘was in a distressed condition’, was ‘perfectly rational but somewhat agitated’ or had a ‘condition … consistent with assault having taken place’.^69^ These statements were brief and no conclusion was attached to them, but this was also the case with most other statements concerning the physical condition of the complainants. This suggests that, before the 1970s, in the majority of cases under review, medical witnesses did not mention emotions, though some forensic physicians considered short-term emotions in rape cases and referred to them as evidence in court.

In the period 1922–1978, mainstream forensic experts did discuss the emotional experiences of rape victims but did not consider them as long-term psychological consequences of rape that required care. Instead, for the authors of the handbooks, the emotion of fear was a piece of evidence. Their main aim was to determine whether the woman in question would have been likely to have experienced it. To do so, they favoured a sceptical attitude over a sympathetic approach. Moreover, in their assessment, they did not rely on knowledge from the psy-sciences but predominantly on moral ideas concerning sexuality. This raises the question of why knowledge of long-term psychological trauma and the necessity of sympathetic care were absent from mainstream forensic literature. The last sections of this article turn to this question.

## Gender and Expert Appointment Practices

Differences in the medical professions influenced whether doctors considered the psychological impact of rape. The doctors who emphasised the long-term mental effects of rape were medical practitioners who encountered victims of sexual violence sometime after the crime had been committed. The doctors of the MWF were psychiatrists, psychotherapists, doctors with a V.D. clinic, G.Ps and gynaecologists. Sometimes they only encountered these victims because these women explicitly sought out their help. These doctors, unlike the forensic physicians who only saw the victim when she made a complaint, were in a position to see the long-term impact of rape. They encountered the women in their caring capacity whereas their colleagues in the context of the police’s request to find evidence. Because the medical procedures connected to criminal investigation were primarily focused on evidence-gathering and not on caregiving practices, therapists were not asked to see victims and did not play a significant role in rape examinations.

In addition to this, the gendered appointment practices of the police obstructed the integration of the doctors from the MWF into forensic procedures. Female physicians, already in short supply during the first half of the twentieth century, were often overlooked by the chief constables of the local police stations who had the right to decide who would examine victims of sexual violence. These men often preferred to appoint their police surgeon or a doctor already familiar to them, while dismissing female doctors. A telling example is a case from 1925 when the police station in Plymouth actively prevented a woman doctor from examining a rape victim. It concerned a 16-year-old girl who was 5–6 months pregnant and admitted to a Salvation Army Home. The police contacted the matron of the home because they wanted the girl examined. The matron asked whether the girl would be examined by a woman doctor and ‘after some hesitation was informed by the police “no”’. The matron refused to let the girl go and argued that the woman doctor who visited the home, Dr Ramsay, should examine her. The police answered that they wanted to spare Dr Ramsay the trouble of going to the assizes. Yet, when Dr Ramsay said she was prepared to appear in court, the police refused her services and threatened that they would come and collect the girl from the Home. To prevent this, the matron sent the girl back to her parents and told them not to have her examined by a male doctor. However, the police contacted the mother and warned her that if she wanted the child to be examined by another doctor, she would have to pay the expenses. Eventually, the stepfather yielded and had her examined by a prison doctor, as the police wished. Dr Ramsay brought this case to the attention of the MWF and the Ministry of Health. Yet, from the documentation, it seems unlikely that the matter was ever pursued. Responding to her letter, Janet M. Campbell wrote that she went to see Sir Blackwell of the HO about the case and that he was going to ask the police for a report on it. However, she finishes her letter with ‘but I don’t think, between ourselves, that you will get much satisfaction from the HO’.^70^

The situation did not improve much over the next decades. Though Nesta Wells was appointed as ‘woman police surgeon’ in Manchester in 1927, for years she remained the only one in the country while other police districts refused to see the value of employing a woman doctor.^71^ In Doncaster, in 1938, the health committee had asked a woman doctor, Betty Walker, to undertake the examinations in cases of sexual assault. Yet, in practice, police refused her the title of woman police surgeon and rarely referred cases to her because the chief medical officer, responsible for public health, ‘felt that if [her] name was officially associated with the police, this might deter patients from attending my clinics, particularly the V.D. Clinic’.^72^ Another example came from 1942 Bradford, where the chief constable argued that there was no need for a woman doctor, as he recently employed twenty-one policewomen who did not require the service of a medical woman. Even though it was pointed out to him that the woman doctor would care for young victims of sex crimes, he did not see the difference between the needs of his personnel and women and children who experienced sexual violence.^73^ The fact that the matter of employing women police surgeons emerged again in the 1980s, illustrates the difficulty these women had in claiming their position in the forensic landscape.^74^

In sum, the doctors who voiced concern for the mental health of rape victims were not involved in, or even actively shunned from the medico-legal procedures by the police. Still, their absence in forensic practices only partly explains why knowledge on long-term psychological consequences of rape and the value of a sympathetic approach were absent from the handbooks. To understand this, the next sections turn to the epistemic virtues that shaped British forensic culture.

## Sympathy Versus Impartiality

The MWF advocated that rape victims should be approached sympathetically because of the mental strain they experienced. Nevertheless, during most of the twentieth century, sympathy was not considered a forensic virtue. Though some experts considered the display of sympathy a useful tool for extracting knowledge from victims, many feared that expressing true feelings of sympathy and paying attention to the emotional experience of the victims would prevent them from upholding one of the key virtues in forensic science and medicine: impartiality.

Dr Bourne’s case shows that many physicians considered a doctor’s attention to trauma to be inextricably bound up with a sympathetic, and hence partial, approach. According to newspaper articles and journal reports on the case, the prosecution, police officers, and some medical practitioners criticised Bourne for letting his feelings interfere with his practice. But Bourne and his defence lawyer explicitly denied that he had merely acted out of ‘pity’ or sympathy.^75^ Instead, Bourne tried to present himself as a brave pioneer who had willingly given himself up for arrest and brought forward a ‘test case’ in order ‘to establish in the eyes of the law that mental health was just as important as physical health’.^76^ However, even when he was acquitted, some of his colleagues argued that his attention to mental health could not be distinguished from his subjective feelings of sympathy for the victim. In a letter to the editor of the *BMJ*, one general practitioner complained that ‘Mr. Bourne acted ab initio on purely sentimental, moral, and sympathetic grounds’.^77^ And another colleague remarked that ‘it can hardly be in doubt that … the obstetrician is sometimes influenced by humanitarian considerations in performing abortion on such ravished children’.^78^ Attention to psychological well-being was closely connected to the doctor’s sentiment and sympathy.

The struggle of expert witnesses to present themselves as impartial knowledge-makers made them adopt an unsympathetic attitude towards victims. In the modern British legal arena, expert witnesses were called into court by either the prosecution or defence party. This meant that either one of these parties employed the expert and that during the trial, the expert appeared for them in the courtroom and was cross-examined by the opposing party. In this adversarial system, experts were vulnerable to accusations of partiality. The advice literature on forensic medicine and science illustrates that forensic experts were insecure about their position in the British legal system and stressed their need to embody the legal virtue of impartiality to counter suggestions of bias.^79^ Court advice columns warned expert witnesses that ‘bias and sympathy on the doctor’s part may easily lead to an impulsive and rash opinion’.^80^

To legitimise their expert role, authors of forensic handbooks presented themselves as particularly capable of avoiding this pitfall. The 1948 edition of *Taylor’s Principles*—a leading book on medical jurisprudence—pointed out that the crime which ‘is so rightly detested’ required ‘great circumspection’ on behalf of the medical examiner because ‘when a girl over sixteen or a woman is in question, juries are very inclined to take the view that there cannot be smoke without fire’.^81^ It became the duty of the medical examiner ‘to exculpate an unjustly accused man’ and remain untouched by these lay feelings of sympathy.^82^ Various authors of forensic textbooks and articles noted that this was how the forensic expert distinguished himself from a family doctor. A divisional police surgeon for the Merseyside Police explained ‘in the doctor’s surgery, the doctor tends to accept most of what his patient tells him as the truth. In the police station, no such assumption can be made and allegations of assault can be made maliciously by people of the most innocent appearance’.^83^ It was a mark of forensic expertise to be able to switch off this ‘natural feeling’ of sympathy and instead adopt a critical attitude towards the victim.

This dismissive attitude towards the virtue of sympathy would have complicated the attempt of women doctors to carve out a position for themselves in the forensic landscape. In the early and mid-twentieth century, medical women, in their quest for emancipation, had pointed to their ‘natural’ feminine qualities of care and sympathy to legitimise their presence in the medical profession.^84^ But the association between women and sympathy would not have aided the MWF’s plea for employing more women police surgeons. It was a trait more associated with nurses than doctors.^85^ Moreover, many police officers believed that the role of a sympathetic woman could also be played by a policewoman or social worker, making it unnecessary to employ female doctors. This changed after the 1970s when feminist activism called for equality in the workplace and public criticism of the unsympathetic attitude of police surgeons was uttered. As larger numbers of women made their way into the mainstream police force and forensic services, the discussion of whether rape victims should be examined by women police surgeons flared up again. However this time, the argument that ‘a sympathetic male doctor is just as good’ was used to counter their claim.^86^

After 1978, handbooks pointed to the importance of approaching a rape victim with sympathy and paying attention to her mental health. It seems that sympathy had become a mainstream forensic virtue. Still, while on paper many police surgeons emphasised the importance of a sympathetic approach, in practice, forensic physicians kept struggling to combine a sympathetic approach with an impartial attitude. In the courtroom, experts would continue to ‘be accused of lacking neutrality and working for the prosecution’.^87^ In fact, one doctor working in the 1990s ‘recalled how she had been accused by the defence of having “emotionally bonded to the victim” because she had treated her with sympathy’.^88^

A sympathetic approach clashed with the attempts of expert witnesses to embody the virtue of impartiality. An epistemic virtue better suited for the enactment of impartiality was detachment.

## Detachment in Twentieth-Century British Forensic Culture

British forensic experts attached much importance to detachment because it was embedded in the modern medical and British emotional practice of the early and mid-twentieth century. Around the eighteenth century, growing importance was placed on anatomy and pathology in medicine: to understand the body, it was opened up, dissected, and individual organs were studied.^89^To execute this kind of work, pathologists adopted a stoical perspective on the bodies they dissected and came to see them primarily as objects of study.^90^ This approach was not contained within the walls of the mortuary but came to influence modern medical practice in the late nineteenth and early twentieth centuries.^91^ In this dominant ‘anatomy-lab culture’, physicians came to see their patients less as subjects with emotional needs and more as objects of study which had to be approached in a detached manner.^92^While this practice was critiqued publicly, British forensic practice, where the pathologist was one of the most influential figures, proved fruitful soil for this medical culture.^93^

The handbooks that advised forensic physicians on rape examinations were written by eminent forensic experts, often men, who enjoyed a popular status in Britain as medical and scientific investigators—such as Sydney Smith, John Glaister Jr and Keith Simpson. These were trained pathologists, who predominantly worked with dead bodies. They wrote about medico-legal matters from this point of view. The autobiographies written by these experts testify that detachment was one of the core forensic virtues they sought to embody in the mid-twentieth century. Almost all experts who wrote memoirs stressed that the job did not affect them on an emotional level because they were particularly good at distancing themselves from the bodies they encountered. For example, Simpson remarked in his autobiography in 1980 that ‘even as a young man I felt no emotional disturbance when I performed post-mortems’.^94^ And Glaister reflected in his 1964 memoires that ‘all medical work, whether pathological or operative, was a realm of work … a place which must be dissociated from my personal life and emotions’.^95^ To carry out their work forensic pathologists approached the victims of crime not as persons but as study objects to be researched.

Despite their specialisation, these pathologists influenced rape examination practices. That is because, until the last decades of the twentieth century, the handbooks and the lectures of these experts were the primary sources of information on all medico-legal matters, including rape. In early and mid-twentieth-century Britain, rape victims were examined by a registered medical practitioner consulted by the police. This did not have to be a specialist but was often a police surgeon or general practitioner. These physicians relied on the, often non-mandatory, lectures on forensic medicine they had attended during their studies and on the handbooks written by pathologists who considered themselves all-round experts. In this light, it seems accurate that one rape victim described that during the medical examination, it was ‘as if I was on the slab in the morgue’.^96^

So while detachment was shaped through pathologists’ interactions with corpses, this virtue also influenced rape examinations. This is illustrated, for example, in the correspondence about the resignation of the Police Surgeon of Aberdeen, Dr Alexander Brown. On 17 February 1931, Dr Brown wrote to the British Medical Association to explain why he was abandoning his post. One of his main reasons for leaving the job was the mental impact examining rape victims had on him. He explained that when he was appointed he was made to believe that he would be primarily conducting ‘malingering cases’ but in reality, he primarily worked at night and those ‘night calls [we]re usually criminal attacks on young girls, sometimes mere infants and are of the most revolting nature’.^97^He ended his letter by emphasising two times that ‘the figures given are merely an index of the amount of work done. They convey nothing of the responsibility involved or of the disgusting nature of much of the work’.^98^ The British Medical Association discussed the matter and the medical secretary wrote that he would look into most of the grievances Dr Brown outlined but added

I should not myself be inclined to place as much stress as Dr Brown does on what he calls the ‘disgusting nature’ of some of the cases he has to attend to. Surely that could be said by every doctor with regard to quite a fair proportion of his work, and as every medical student is taught in hospital to regard the repugnant things which come his way as being part of the day’s duty, I should be inclined to deal with that aspect only en passant.^99^

In this rare event that a doctor addressed the particular emotional strain of forensic examinations, this experience was downplayed by the Medical Association by pointing to the need of every doctor to conform himself to the emotional virtue of detachment.

Because of the hold of the virtue of detachment over forensic experts, there are few traces of the emotions of examiners in the archives. Still, other medical practitioners also expressed affect in rape examinations. For example, in 1938, an enthusiastic divisional surgeon, Dr Grosky, had offered to provide lectures for police officers on medico-legal subjects but this proposal was considered inappropriate by one of his colleagues, Dr Jones. Jones stressed ‘the very real dangers of young constables knowing too much on these repulsive medical subjects’.^100^ And in 1942, Superintendent Book was paraphrased to have said that ‘the men doctors disklike “carnal knowledge” cases’.^101^ Even pathologist Dr Keith Simpson admitted to having an emotional reaction to the rape victim of the ‘A6 Murder’—a case from 1961 in which a man first shot the driver of a car and afterwards raped and shot the driver’s girlfriend who, unlike her boyfriend, survived the shooting. He writes that when he examined her ‘I felt (as I often did) more disturbed at this tragic sight in a living creature than by a dead body’.^102^ This was an unusual remark for him, as he presented himself as a forensic pathologist who was seldom emotionally triggered by his work and whose emotions never influenced his knowledge-making process. According to psychiatrist Edward Glover, medical men had difficulty being objective in sexual offences cases. In a lecture to the medico-legal society on 24 May 1945, he stated that ‘we might as well begin with the frank admission that few, if any, of us can approach the problem of sexual disorder with *that complete emotional detachment* that is the pre-requisite of successful research’.^103^

These small glimpses into the emotional life of medical examiners involved with rape victims illustrate that even though experts were not keen to admit emotional involvement, rape cases, struck a nerve. Those admissions are scarce. The scarcity of such reflections on the emotional experiences of forensic examiners in rape cases points less to the absence of emotions and more to how unusual it was for experts to acknowledge and express them. Still, their presence does not mean that a sympathetic medical practice was in place. For this to develop emotions do not only have to be present but also acknowledged.^104^ And as the reply to Dr Brown’s resignation letter and Glover’s critique of his colleagues show, the culture of detachment required doctors to turn their attention away from the emotions they felt and witnessed in sexual crimes.

The practice of distancing oneself from emotions would have been particularly strong in British forensic culture. In early and mid-twentieth-century Britain, control over emotional display was considered very important. Historians of emotion in Britain argue that ‘rigid emotional control’ in public increasingly became a British attribute in the nineteenth and twentieth centuries.^105^ In the years during and after the World Wars, the emotional practice that became dominant was that of the ‘stiff upper lip’, a stoic attitude typical for the British who valued a display of self-control in the light of emotionally demanding situations.^106^ The immense scale of loss during the wars changed public ideas about emotion, and public expressions of grief and bereavement were increasingly considered inappropriate and unpatriotic in the UK.^107^ While the ‘stiff-upper-lip’ was a general British ideal, especially men were expected to hide emotions to ‘demonstrate the masculine virtue of self-control’ as emotional expressiveness continued to be associated with femininity until at least the 1960s.^108^To sum up, in mainstream forensic literature—dominated by male pathologists—there was little room for sympathy or attention to the emotional experiences of rape victims that required care because of the prominent role that the virtue of emotional detachment played in British forensic culture.

## Conclusion

In the period 1924–1978, the emotions of rape victims were an important theme in British forensic culture. Outside mainstream forensic literature, medical practitioners emphasised the long-term psychological impact that the experience of rape could have on victims, especially children. The cases of the MWF and Dr Bourne illustrate that knowledge of long-term mental suffering circulated inside the forensic context decades before the 1970s. Some of these physicians, predominately women doctors, argued that a sympathetic approach on behalf of the examiner could lighten the mental strain of the victim.

Yet, these views were not expressed in mainstream forensic literature. Here, the emotional experiences of rape victims were not considered as a mental health issue but as pieces of evidence. Specifically, forensic physicians looked for signs of fear which could have driven the victim into a state of physical paralysis, hence explaining the absence of injuries medical practitioners expected to find in a rape victim. In their assessment of the mental condition of the victim, forensic experts did not rely on psychological theories on the victim’s responses to rape. Nor did they normally advocate a sympathetic and caring approach to lessen the victim’s emotional burden. Instead, they advocated a critical, even stoical attitude, to assess the ‘credibility’ of the girl or woman. This article has argued that this detached approach towards the emotions of rape victims remained mainstream in forensic medicine until the 1970s.

To understand why this was the case, I brought together police practice, epistemic virtues and emotional experiences of professionals. Applied to the history of rape examinations this shows that historians benefit from considering how knowledge practices were shaped by gender prejudices and epistemic virtues.

The medical women who advocated a sympathetic approach with attention to psychological trauma in victims were less influential in the forensic realm than their male counterparts, as the police often refused to assign cases to them. The virtue of sympathy and practices of care they propagated were associated with femininity and not taken up in the male-dominated field of forensics.

Moreover, forensic doctors associated their job with knowledge-making rather than caring practices. Expert witnesses feared that a sympathetic approach was incompatible with the judicial virtue of impartiality, which they had to embody to be taken seriously in court. Impartiality matched better with the virtue of detachment which formed a key characteristic of modern medical culture and resonated with the British and masculine ideal of the ‘stiff-upper-lip’. Detached from their patient’s emotions and their own, forensic experts approached rape victims and their emotions as objects to study and not as emotional patients whose psychological problems they had to tend to. It required forensic practitioners’ introspection and reflection upon a public outcry for sympathy to change this perspective later in the twentieth century.

## Footnotes

1 I will use the term victim–survivor when addressing contemporary debates to acknowledge the debate on the terminology but I will use the term victim when referring to historical cases as not to employ the concept anachronistically. For more information on this issue see: Joanna Bourke, *Disgrace: Global Reflections on Sexual Violence* (Pittsburgh: Reaktion Books, 2022), 18–20.

2 Josephine Burnell Howitt and Deborah Rogers, ‘Adult Sexual Offences and Related Matters’, in W. D. S. McLay and Association of Police Surgeons, eds, *Clinical Forensic Medicine* (London: Greenwich Medical Media, 1996), 193–218, 201.

3 Maria Nittis, ‘Sexual Assault Examination: A Physician’s Guide’, in Margaret M. Stark, ed, *Clinical Forensic Medicine: A Physician’s Guide* (Cham: Springer International Publishing, 2020), 63–142, 65.

4 Or at least, that is the ideal. As Joanna Bourke argues, in the twenty-first century ‘complaints about the medical treatment of rape victims continue to be heard’ and ‘major grievances about medical examinations are routine’. Joanna Bourke, ‘Police Surgeons and Victims of Rape: Cultures of Harm and Care’, *Social History of Medicine*, 2018, 4, 711–31, 729.

5 Joanna Bourke, ‘Sexual Violence, Bodily Pain, and Trauma: A History’, *Theory, Culture & Society*, 2012, 3, 25–51, 26–28.

6 Michal Shapira, *War Inside: Psychoanalysis, Total War, and the Making of the Democratic Self in Postwar Britain* (New York: Cambridge University Press, 2013), 21.

7 Adrian Bingham et al., ‘Historical Child Sexual Abuse in England and Wales: The Role of Historians’, *History of Education,* 2016, 4, 411–29; Bourke, ‘Sexual Violence, Bodily Pain, and Trauma’; Louise A. Jackson, *Child Sexual Abuse Victorian England* (London: Routledge, 2000), 135–38.

8 Bourke, ‘Sexual Violence, Bodily Pain, and Trauma’, 28.

9 Other historians have already pointed out that the (short-term) psychological and emotional impact of rape was already discussed in court cases and forms of life-writing. See: Victoria Bates, ‘Forensic Medicine And Female Victimhood In Victorian And Edwardian England’, *Past & Present*, 2019, 1, 117–51; Willemijn Ruberg, ‘Trauma, Body, and Mind: Forensic Medicine in Nineteenth-Century Dutch Rape Cases’, *Journal of the History of Sexuality*, 2013, 1, 85–104; Lucy Delap, ‘“Disgusting Details Which Are Best Forgotten”: Disclosures of Child Sexual Abuse in Twentieth-Century Britain’, *Journal of British Studies*, 2018, 1, 79–107.

10 Louise A. Jackson, ‘Women Professionals and the Regulation of Everyday Violence in Interwar Britain’, in Shani D’Cruze, eds, *Everyday Violence in Britain, 1850–1950: Gender and Class* (Essex: Pearson Education Limited, 2000) 119*–*35.

11 Katherine D. Watson, *Medicine and Justice: Medico-Legal Practice in England and Wales, 1700–1914* (Abingdon: New York, 2019), 19; Ian Burney and Neil Pemberton, *Murder and the Making of English CSI*, (Baltimore: Johns Hopkins University Press, 2016), 41.

12 Jennifer Temkin, ‘Medical Evidence in Rape Cases: A Continuing Problem for Criminal Justice’, *The Modern Law Review* 1998, 6, 821–48; Bourke, ‘Police Surgeons and Victims of Rape’.

13 Bourke, ‘Police Surgeons and Victims of Rape’, 718.

14 Ibid., 718.

15 Victoria Bates, *Sexual Forensics in Victorian and Edwardian England: Age, Crime and Consent in the Courts* (Bashingstoke: Palgrave Macmillan, 2016); Victoria Bates, ‘“Under Cross-Examination She Fainted”: Sexual Crime and Swooning in the Victorian Courtroom’, *Journal of Victorian Culture,* 2016, 4, 456–70; Joanna Bourke, *Rape: A History From 1860 To The Present* (London: Virago, 2007); Ivan Crozier and Gethin Rees, ‘Making a Space for Medical Expertise: Medical Knowledge of Sexual Assault and the Construction of Boundaries between Forensic Medicine and the Law in Late Nineteenth-century England’, *Law, Culture & the Humanities*, 2012, 2, 285–304; Jennifer Temkin, Jacqueline M. Gray, and Jastine Barrett, ‘Different Functions of Rape Myth Use in Court: Findings From a Trial Observation Study’, *Feminist Criminology*, 2018, 2, 205–26; Shani D’Cruze, ‘Sexual Violence since 1750’, in Sarah Toulalan and Kate Fisher, eds, *The Routledge History of Sex and the Body: 1500 to the Present* (London: Routledge, 2013), 444–59.

16 Keith Mant, *Forensic Medicine: Observation and Interpretation* (London: Lloyd-Luke, 1960), 230.

17 Bourke, ‘Police Surgeons and Victims of Rape’, 730.

18 Graham Matthews, ‘Clinical Empathy and the Ethics of “Detached Concern” in Mid-Twentieth-Century British Literature’, *Literature and Medicine*, 2020, 1, 113–40; Joanna Bourke, ‘Pain, Sympathy and the Medical Encounter between the Mid Eighteenth and the Mid Twentieth Centuries’, *Historical Research: The Bulletin of the Institute of Historical Research*, 2012, 229, 430–52.

19 Burney and Pemberton, *Murder and the Making of English CSI*.

20 Aleck Bourne, *A Doctor’s Creed: The Memoirs of a Gynaecologist* (Hertfordshire: The Garden City Press Limited, 1962), 99; Aleck Bourne, ‘Abortion and the Law: Correspondence’, *British Medical Journal*, 1938, 2, 254.

21 Bourne, ‘Abortion and the Law’, 254.

22 ‘Charge of Producing Abortion: Mr Bourne Acquitted’, *British Medical Journal,* 1938, 2, 200.

23 ‘Trooper Thomas Guilty of Serious Offence’, *Shields Daily News,* 28 June 1938, 1.

24 Ibid., 1.

25 ‘Charge of Producing Abortion’, 200.

26 Ibid., 200.

27 Ibid., 199.

28 Bonnie Evans, *The Metamorphosis of Autism: A History of Child Development in Britain* (Manchester: Manchester University Press, 2017); Juliana Broad, ‘Working in Cases: British Psychiatric Social Workers and a History of Psychoanalysis From the Middle, C.1930–60’, *History of the Human Sciences* 2021, 34, 169–94.

29 Ibid., 202.

30 Ibid.

31 Ibid.

32 Ibid., 203.

33 Sexual Offences Against Young Persons, *The Medical Officer*, Home Office Committee to Enquire into Sexual Offences against Young Persons, 8 March 1925, SA/MWF/D.7, Medical Women’s Federation, Wellcome Collection, London. (Henceforth Home Office Committee Sexual Offences).

34 Violet Kelynack, Letter to Home Office by Medical Women Federation, June 1938, SA/MWF/D.18, Women Police Surgeons, Wellcome Collection, London (Henceforth Women Police Surgeons).

35 Bourke, ‘Sexual Violence, Bodily Pain, and Trauma’, 40.

36 Willemijn Ruberg, ‘Trauma, Body, and Mind: Forensic Medicine in Nineteenth-Century Dutch Rape Cases’, *Journal of the History of Sexuality,* 2013, 1, 85–104, 102–103; Bates, *Sexual Forensics*, 134.

37 Dr Erie Evans, Home Office Committee Sexual Offences.

38 Notes are not dated but the other letters and documents suggest that they were written in 1942.

39 Dr Elspeth Macleod, Women Police Surgeons.

40 Nesta Wells and Basil Henriques, ‘Sexual Assaults on Children’, *The British Medical Journal*, 1961, 2, 1629–33, 1633.

41 Jackson, ‘Women Professionals’, 127.

42 Barbara Reeve, Women Police Surgeons.

43 Shapira, *War Inside*, 9.

44 Ibid., 21.

45 Joanna Bourke, ‘Forensic Sense: Sexual Violence, Medical Professionals and the Senses’, in Rob Boddice and Bettina Hitzer, eds, *Feeling Dis-Ease in Modern History: Experiencing Medicine and Illness*, (London: Bloomsbury Collections, 2022), 36.

46 Jackson, Women Professionals, 120.

47 Ibid., 124–25.

48 Anne Digby, *The Evolution of British General Practice: 1850–1948* (Oxford: OUP Oxford, 1999), 185.

49 Mabel Lindsey, Women Police Surgeons.

50 Note, there were also some women doctors who used a less compassionate, even distrusting, discourse when discussing these matters.

51 Miss Beattie, Women Police Surgeons.

52 Janet M. Campbell, Women Police Surgeons.

53 Douglas J. A. Kerr, *Forensic Medicine: A Textbook for Students and a Guide for the Practitioner* (London: A&C Black LTD, 1935), 163.

54 W. G. Aitchison Robertson, *Aids to Forensic Medicine and Toxicology* (London: Baillière, Tindall and Cox, 1922) 56–57.

55 John Glaister, *Medical Jurisprudence and Toxicology* (Edinburgh: E. S. Livingstone, 1942), 354.

56 Dixon Mann, *Forensic Medicine,* 79.

57 Sydney Smith, *Forensic Medicine: A Textbook for Students and Practitioners* (London: J. & A. Churchill, 1925), 220.

58 Frederick J. McCann, ‘Some Cases of Medico-Legal Interest in the Practice of Gynaecology’, *Transactions of the Medico-Legal Society,* 1926–1927, 83, 83–102; Sydney Smith, W.G.H. Cook, and C.P. Stewart, *Taylor’s Principles and Practice of Medical Jurisprudence* (London: J. & A. Churchill, 1948) 83.

59 ‘Forensic Medicine Lecture Notes’, October 1932, Papers of John Brash De Vere Weir, GB 248 DC 206/7/12 and DC 206/7/13, Glasgow University Archive.

60 Mathew Thomson, *Psychological Subjects: Identity, Culture, and Health in Twentieth-Century Britain: Identity, Culture, and Health in Twentieth-Century Britain* (Oxford: OUP Oxford, 2006), 175, 182, 195.

61 Frederic Smith, *London Hospital Lectures on Forensic Medicine and Toxicology* (London: H. K. Lewis & Co. LTD., 1929); Kerr, *Forensic Medicine*, 165; Caryl Thomas, *A Synopsis of Forensic Medicine and Toxicology* (London: J. Wright, 1933), 80–83; Simpson, *Forensic Medicine*, 184; Smith, Cook, and Stewart, *Taylor’s Principles*, 78; Glaister, *Medical Jurisprudence and Toxicology*, 354; Francis E. Camps, ‘The Medico-Legal Expert’, *Medicine, Science and the Law* 1968, 1, 11–14; S.H. Burges, ‘Sexual Offences’, in S. H. Burges and J. E. Hilton, eds, *The New Police Surgeon; A Practical Guide to Clinical Forensic Medicine*, (London: Hutchinson Benham Limited, 1978), 230. An exception is Keith Mant who briefly mentions changes in behaviour in girls aged between 11 and 13: Mant, *Forensic Medicine*, 237.

62 Central Criminal Court: Depositions: Charge of Violent Carnal Knowledge, 10 February 1931, Crim 1/542, National Archives Kew (NA), London.

63 Mant, *Forensic Medicine*, 232–33.

64 Ibid., 244; Mann, *Forensic Medicine*, 79.

65 Simpson, *Forensic Medicine*, 184.

66 Ibid., 183.

67 Mann, *Forensic Medicine*, 82; Kerr, *Forensic Medicine*, 164.

68 A Stuart, book of lecture notes forensic medicine, lecture 16, 1921, EUA CA2/7/3, Edinburgh University CRC archive.

69 Central Criminal Court: Depositions: Charge of Rape, 11 December 1928 CRIM 1/451, NA; Central Criminal Court: Depositions: Charge of Violent Carnal Knowledge, 10 February 1931, CRIM 1/542, NA; Central Criminal Court: Depositions: Charge of Rape, 6 January 1959, CRIM 1/3106, NA; Assizes: Western Circuit: Criminal Depositions and Case Papers: Rape, 9 May 1960, ASSI 26/210, NA.

70 Janet M. Campbell, Letter Titled Personal, 23 June 1925, Home Office Committee Sexual Offences.

71 ‘Woman Police Doctor’, *Vote*, 5, 1927, 3; Jackson, ‘Women Professionals’, 119.

72 Betty Walker, Women Police Surgeons.

73 D. Pearson, Women Police Surgeons.

74 Appointment of women police surgeons for examining victims of rape and sexual assault, January 1 1983–31 December 1987, HO 287/4027, NA.

75 ‘Girl’s Chance of Lifelong Mental Injury’, *Leicester Evening Mail*, 18 July 1938; ‘Surgeon Found “Not Guilty”’, *The Times*, 20 July 1938; Bourne, *A Doctor’s Creed*, 99.

76 Bourne, ‘Abortion and the Law’, 254.

77 Charles Franklyn, ‘Correspondence’, *British Medical Journal*, 1938, 2, 373.

78 James Young, ‘Correspondence’, *British Medical Journal*, 1938, 2, 373.

79 ‘The Expert Witness’, *The Lancet*, 1923, 202, 991; ‘Medicine and the Law: Judicial Views of Medical Evidence’, *The Lancet*, 1941, 237, 425; ‘The Duties of the Medical Witness III’, *British Medical Journal*, Medico-Legal, 1934, 1, 600–601; H.A. Burridge, *An Introduction to Forensic Medicine for Medical Students and Practitioners* (London: H.K. Lewis & Co, 1924) 23; Simpson, *Forensic Medicine,* 211.

80 ‘Doctor in the Box’, *The Lancet*, 274, 1959, 838.

81 Smith, Cook, and Stewart, *Taylor’s Principles*, 94.

82 Ibid., 94.

83 M. Clarke, ‘Examination of the Living’, in *The New Police Surgeon: A Practical Guide to Clinical Forensic Medicine*, ed. S.H. Burges and J.E. Hilton (London: Hutchinson Benham Limited, 1978), 118.

84 Digby, *The Evolution of British General Practice*, 174; Jackson, ‘Women Professionals’, 120 and 122.

85 Sarah Chaney, ‘Before Compassion: Sympathy, Tact and the History of the Ideal Nurse’, *Medical Humanities* 2021, 4, 475–84.

86 ‘Male or Female? Sex Assault Examiners’, *The Police Surgeon Supplement*, 1983, 15, 30–31.

87 Temkin, ‘Medical Evidence’, 840.

88 Ibid.

89 Willemijn Ruberg, *History of the Body* (London: Palgrave Macmillan/Red Globe Press, 2020).

90 Samuel J. M. M. Alberti, *Morbid Curiosities: Medical Museums in Nineteenth-Century Britain* (Oxford, New York: Oxford University Press, 2011), 102.

91 Michael Brown, *Emotions and Surgery in Britain, 1793–1912* (Cambridge: Cambridge University Press, 2022), 17–20.

92 Kelly Underman, *Feeling Medicine: How the Pelvic Exam Shapes Medical Training* (New York: NYU Press, 2020). Sarah-Maria Schober, ‘Muck, Mummies and Medicine: Disgust in Early Modern Science’, *Emotions: History, Culture, Society*, 2020, 4, 43–65; Barbara Duden, *The Woman beneath the Skin: A Doctor’s Patients in Eighteenth-Century Germany* (Cambridge: Harvard University Press, 1998).

93 Agnes Arnold-Forster, ‘Racing Pulses: Gender, Professionalism and Health Care in Medical Romance Fiction’, *History Workshop Journal*, 2021, 91, 157–81.

94 Keith Simpson, *Forty Years of Murder: An Autobiography* (London: Harper Collins, 1980), 380.

95 John Glaister, *Final Diagnosis* (London: Hutchinson, 1964), 24.

96 Bourke, ‘Forensic Sense’, 164.

97 Alexander Brown, Resignation Letter, 17 February 1931, MP408, Police surgeons correspondence, Wellcome Collection. (Hereafter Police surgeons correspondence)

98 Ibid.

99 Dr M. Connon, Police surgeons correspondence.

100 The dangers of advanced lectures by divisional surgeons on medico-legal subjects’, 28 July 1938, MEPO 2/2668, NA.

101 M. Moore, Women Police Surgeons.

102 Simpson, *Forty Years of Murder*, 163.

103 Emphasis added. Edward Glover, These social and legal aspects of sexual abnormality in: Institute for the Scientific Treatment of Delinquency, originally delivered as a lecture to the medico-legal society on 24 may 1945, GUA FM/6/2/21-22, Glasgow University Archive.

104 Underman, *Feeling Medicine,* 2.

105 Thomas Dixon, Weeping Britannia: Portrait of a Nation in Tears (Oxford: New York, 2015); Hera Cook, ‘From Controlling Emotion to Expressing Feelings in Mid-Twentieth-Century England’, *Journal of Social History* 47, no. 3 (2014): 627–46.

106 Ibid.

107 Lucy Noakes, ‘Gender, Grief, and Bereavement in Second World War Britain’, *Journal of War & Culture Studies*, 2015, 1, 123–45.

108 Daniel J. R. Grey, ‘“Agonised Weeping”: Representing Femininity, Emotion and Infanticide in Edwardian Newspapers’, *Media History*, 2015, 4, 468–80, 471; Lucy Delap, ‘Feminism, Masculinities and Emotional Politics in Late Twentieth Century Britain’, *Cultural and Social History,* 2018, 15, 571–93; Dixon, *Weeping Britannia,* 260.

## Notes

Pauline Dirven is a PhD researcher at Utrecht University working on the research project ‘Forensic Culture. A Comparative Analysis of Forensic Cultures in Europe, 1930–2000’. Her research focuses on forensic culture in twentieth-century England, in particular on embodied performances of forensic expertise. She is one of the editors of, and contributing author to the edited volume Forensic Cultures in Modern Europe published in 2023 by Manchester University Press.

